# Planned use of a novel Elastic Traction Device improves efficiency in colorectal endoscopic submucosal dissection: a propensity-score matched study

**DOI:** 10.1007/s00464-025-11807-0

**Published:** 2025-05-29

**Authors:** Shotaro Ishizaka, Tomoaki Tashima, Tomonori Kawasaki, Masami Sano, Tsubasa Ishikawa, Takahiro Muramatsu, Yuki Shiko, Yohei Kawasaki, Yumi Mashimo, Shomei Ryozawa

**Affiliations:** 1 Department of Gastroenterology, Saitama Medical University International Medical Center, 1397-1 Yamane, Hidaka City, Saitama 350-1298 Japan; 2 Department of Pathology, Saitama Medical University International Medical Center, Hidaka City, Saitama Japan; 3 Department of Biostatistics, Graduate School of Medicine, Saitama Medical University, Moroyama-machi, Saitama Japan

**Keywords:** Endoscopy, Gastrointestinal, Colonoscopy, Neoplasm resection, Traction, Elasticity

## Abstract

**Background:**

Endoscopic submucosal dissection (ESD) for colorectal lesions is more challenging than for other sites, leading to the common use of traction devices to improve submucosal visibility and endoscope maneuverability. This study evaluated the outcomes and efficiency of a novel Elastic Traction Device (ETD), a rotatable and reopenable device, in colorectal ESD.

**Methods:**

We retrospectively analyzed lesions (20–50 mm) resected at Saitama Medical University International Medical Center from July 2022 to January 2024. Lesions were divided into the conventional ESD group (C-ESD) and the ETD group (T-ESD); the T-ESD group was further split into the schedule (S) group, where ETD was planned early, and the rescue (R) group, where continued treatment was difficult. Propensity score matching (PSM) was used to balance baseline factors.

**Results:**

Of 260 lesions, 136 were in the C-ESD group and 124 in the T-ESD group. After PSM, 101 lesions from each group were analyzed. No significant difference was observed in procedure time (47.13 ± 36.86 vs. 52.96 ± 39.17 min, *p* = 0.27) or dissection speed (33.54 ± 21.38 vs. 28.38 ± 18.81 mm^2^/min, *p* = 0.07). Perforation rates were similar (4.0% vs. 5.9%, *p* = 0.51). In the S and R groups (58 and 33 lesions, respectively), procedure time was shorter in the S group (43.93 vs. 70.30 min, *p* < 0.01) with faster dissection (35.80 vs. 20.23 mm^2^/min, *p* < 0.0001). Post-ETD attachment, dissection speed improved significantly (*p* < 0.001).

**Conclusions:**

Although the ETD did not consistently reduce procedure time or improve dissection speed, its planned and early application may offer potential benefits. Further prospective multicenter studies are needed to clarify its optimal role and clinical value in colorectal ESD.

**Graphical abstract:**

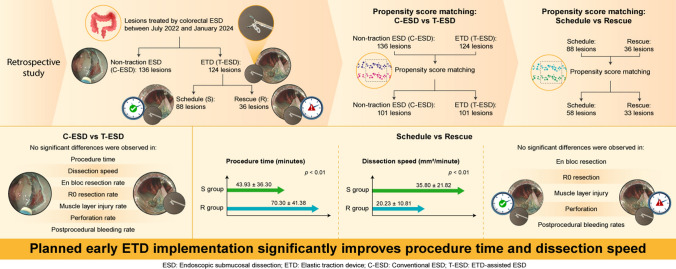

**Supplementary Information:**

The online version contains supplementary material available at 10.1007/s00464-025-11807-0.

Endoscopic submucosal dissection (ESD) has been widely adopted as an effective treatment for superficial colorectal tumors that are difficult to resect en bloc using endoscopic mucosal resection, including adenomas and early colorectal cancer [1, 2]. ESD has also been shown to have a low long-term recurrence rate for these lesions [3]. However, in colorectal ESD, challenges such as poor maneuverability can lead to unsuccessful en bloc resection or complications such as perforation [4]. To overcome the challenges of colorectal ESD, various traction devices have been developed [5–8]. Notably, traction may improve submucosal visibility, allow more efficient dissection, and reduce the risk of complications [9].

The Elastic Traction Device (ETD, MICRO-TECH, Nanjing, China) is a new traction device with a double elastic ring attached to a clip (SureClip, MICRO-TECH, Nanjing, China) that can be easily rotated and reopened. The ETD allows for the readjustment of the target, as well as the direction and strength of the traction [10]. However, reports on the efficient use of ETDs are scarce. Our institution has a uniform treatment strategy for ETDs and believes that planning the use of ETDs before treatment and fitting them as soon as possible would lead to more efficient ETD use. This study aimed to determine the optimal use, efficacy, and safety of ETDs. Identification of the efficient use of ETDs may contribute to improved treatment efficiency and minimally invasive colorectal ESD.

## Materials and methods

This study included lesions treated by colorectal ESD at the Saitama Medical University International Medical Center between July 2022 and January 2024. Indications for ESD were determined based on the colorectal ESD guidelines [11]. Initially, 344 lesions were identified in 339 patients. The following lesions were excluded: nine lesions with traction devices other than the ETD, 32 lesions > 50 mm, 28 lesions < 20 mm, six lesions in which ESD was interrupted because of deep submucosal invasion or strong fibrosis, eight lesions with submucosal tumors, and one lesion with non-neoplastic lesions. Lesions > 50 mm in size were excluded based on previous reports [12, 13]. Colorectal tumors < 20 mm were also excluded because they are not covered by colorectal ESD in Japan [11]. Colorectal ESD was performed by five expert endoscopists and five trainees.

**Fig. 1:**
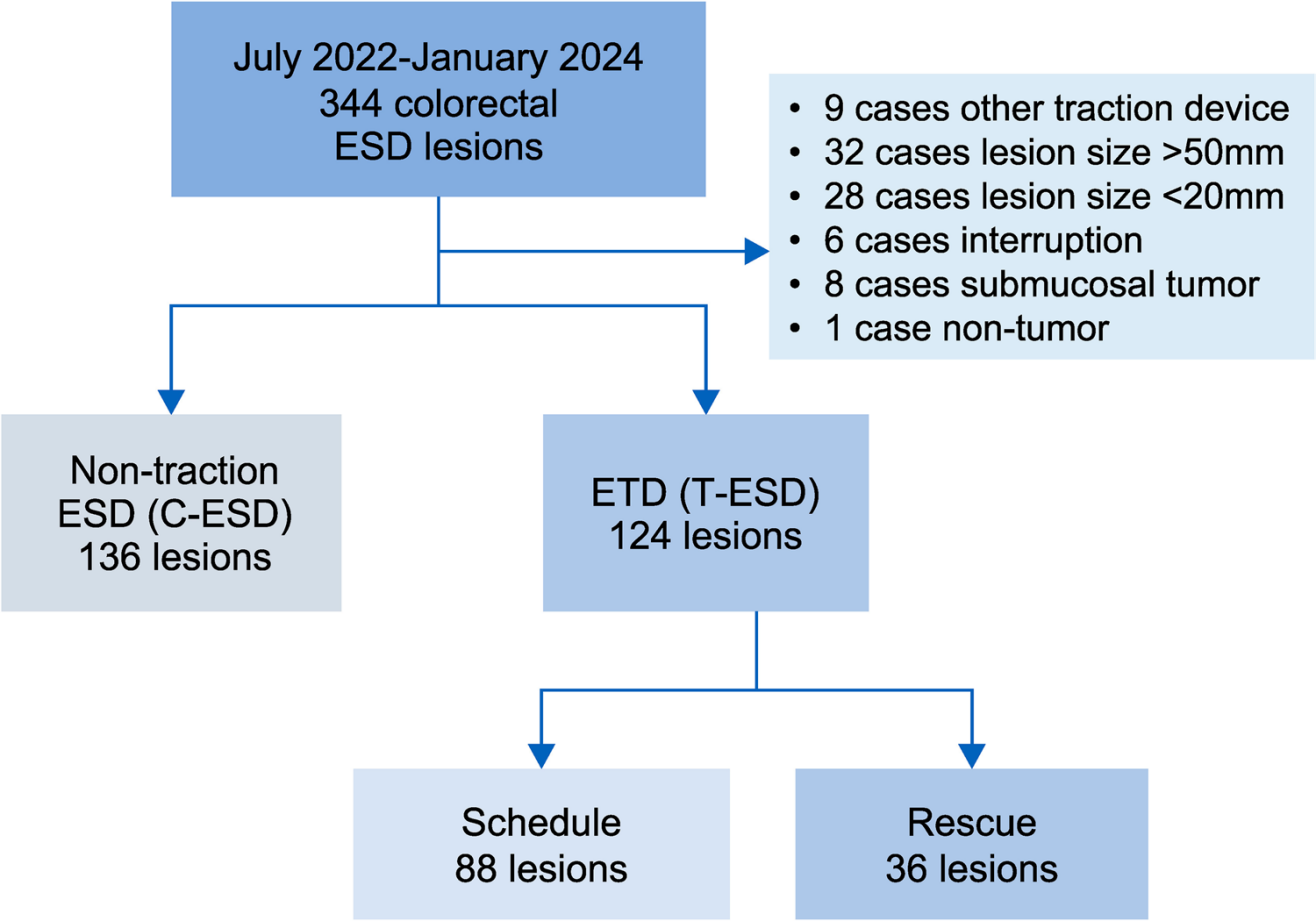
Flowchart of the present study. *ESD* endoscopic submucosal dissection, *ETD* Elastic Traction Device

**Fig. 2:**
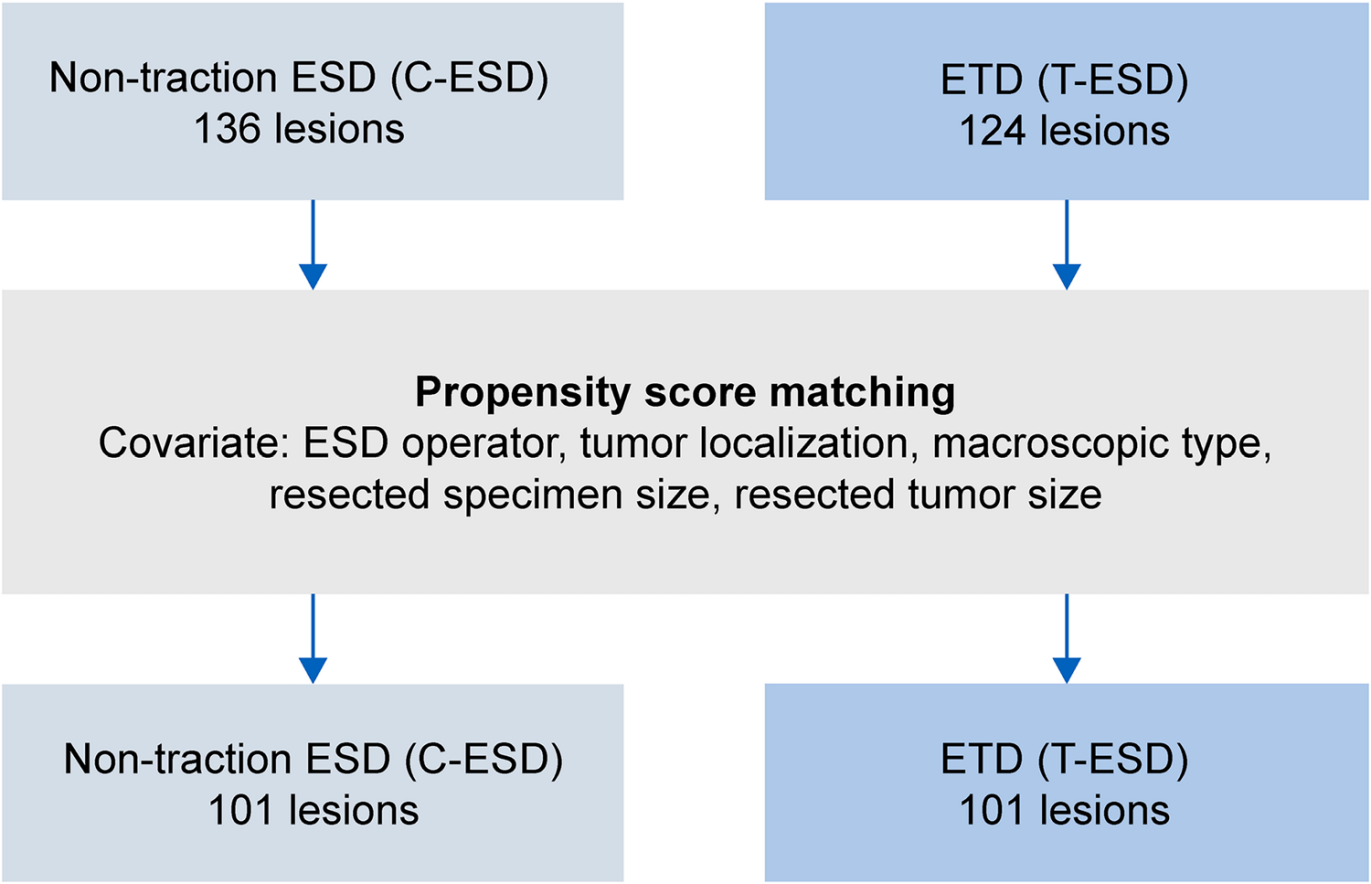
Comparison between the C-ESD and T-ESD groups using propensity score matching. *ESD* endoscopic submucosal dissection, *ETD* Elastic Traction Device

A total of 260 lesions were analyzed. The lesions were divided into the conventional ESD (C-ESD group, 136 lesions) and ETD-assisted ESD (T-ESD group, 124 lesions) groups, and the treatment results were compared (Fig. 1). Propensity score matching (PSM) was applied to adjust for confounders (ESD operator, tumor localization, macroscopic type, resection specimen size, and resected tumor size), and 101 lesions from each group were included in the analysis after PSM (Fig. 2).

**Fig. 3:**
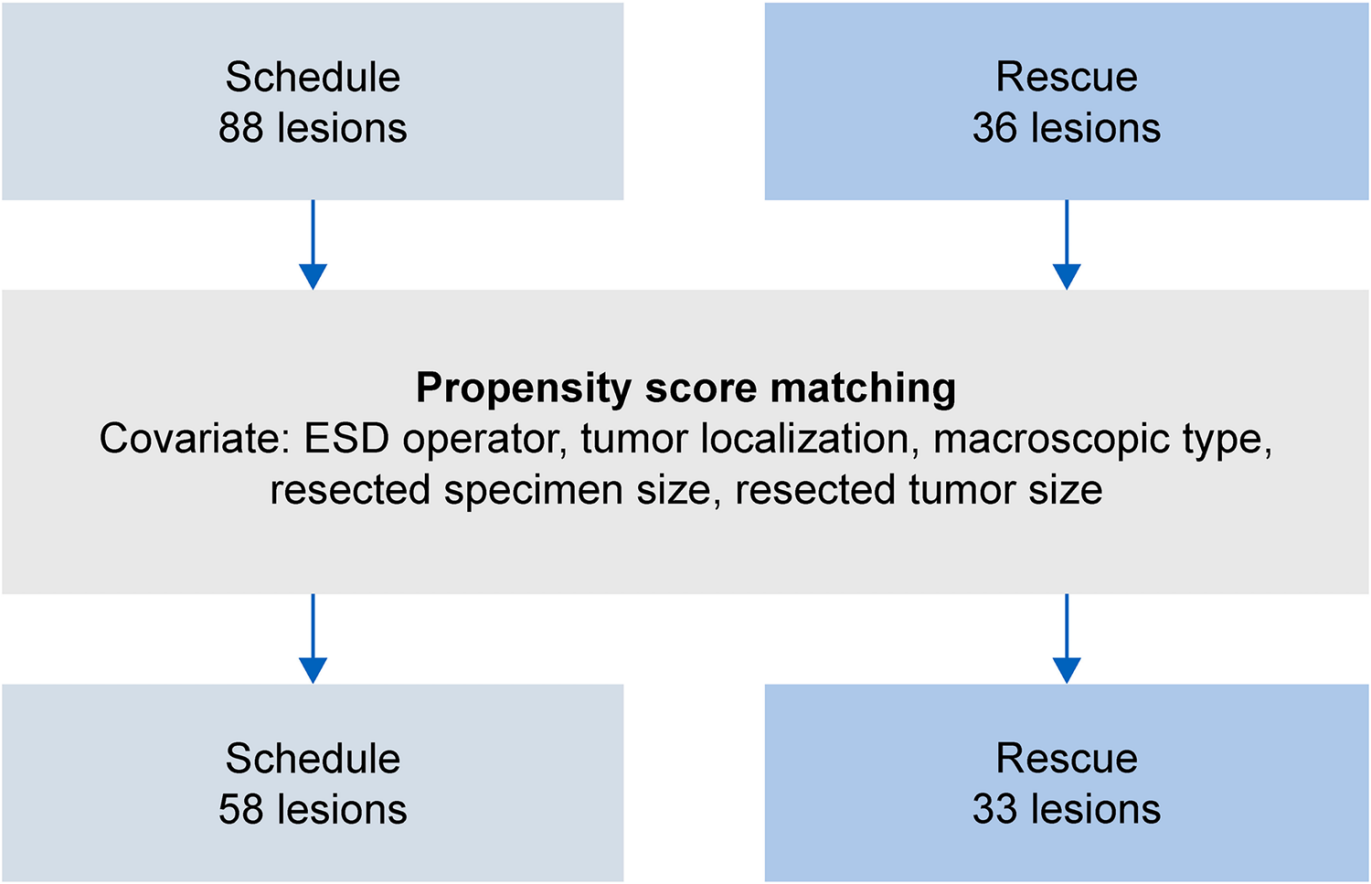
Comparison between the schedule and the rescue groups using propensity score matching

The T-ESD group was further divided into two groups: a scheduled group (S group: 88 lesions), in which ETD use was planned preoperatively and applied as soon as possible, and a rescue group (R group: 36 lesions), in which ETD was used owing to difficulties in continuing the procedure. A detailed comparison was performed using the same PSM approach, with 58 and 33 lesions in the S and R groups, respectively (Fig. 3). The primary outcomes were procedure time and dissection speed, whereas the secondary outcomes included en bloc resection rate, R0 resection rate, and the presence of complications (muscle layer injury, perforation, and delayed bleeding). Additionally, the dissection speeds before and after ETD application in the S group were calculated and compared. The participants were informed in writing, and informed consent was obtained. This study was approved by the Ethics Review Board (IRB) of Saitama Medical University International Medical Center (Institutional ID 20–202).

## The setting of colorectal ESD

Patients received picosulfate sodium hydrate or calcium sennosides A and B orally on the day before the procedure. On the day of the procedure, bowel lavage was performed using sodium and potassium solutions or sodium, potassium, and ascorbic acid solutions. Midazolam was administered intravenously for sedation, and pethidine hydrochloride was used for analgesia. The endoscope used during the procedure was selected by the operator based on the lesion characteristics and ease of manipulation. The endoscopes used were Olympus Medical Systems (Tokyo, Japan), PCF-H290ZI, PCF-H290TI, and GIF-H290TI with a transparent attachment (Elastic Touch, Top Corporation, Tokyo, Japan) on the tip.

The knives used during the procedure were selected by an operator from the Dualknife J (Olympus Medical Systems, Tokyo, Japan), TechKnife (MICRO-TECH, Nanjing, China), and IT-knife nano (Olympus Medical Systems, Tokyo, Japan). The high-frequency generator used was a VIO3 from ERBE (Tübingen, Germany). A mixture of 0.4% indigo carmine solution, purified sodium hyaluronate, and 0.1 mL of adrenaline was used for the local injection.

## ESD procedure

### Conventional ESD

The initial mucosal incision was made on the oral or anal side of the lesion, depending on the morphology of the lesion and the maneuverability of the endoscope after injecting a submucosal injection material at that site. Subsequently, additional mucosal incisions and dissections were performed to create a flap. Next, the mucosal incision was extended, and the submucosal layer of the extended section was dissected. After completing a circumferential mucosal incision around the lesion, the submucosal layer was sequentially dissected using the initially created flap, leading to en bloc resection of the lesion.

### Traction-assisted ESD using ETD

The injection of the submucosal injection material and the mucosal incision prior to ETD placement were performed in the same manner as in conventional ESD. Subsequently, the ETD was attached to the anal or oral side of the lesion at respective time points in the schedule and rescue groups. The ETD is equipped with a silicone band attached to a clip. This device simplifies the setup process because the traction device is already attached to the clip, allowing for easy application. In addition, the ability to rotate and reopen allows precise placement without grasping the fascia propria.

**Fig. 4:**
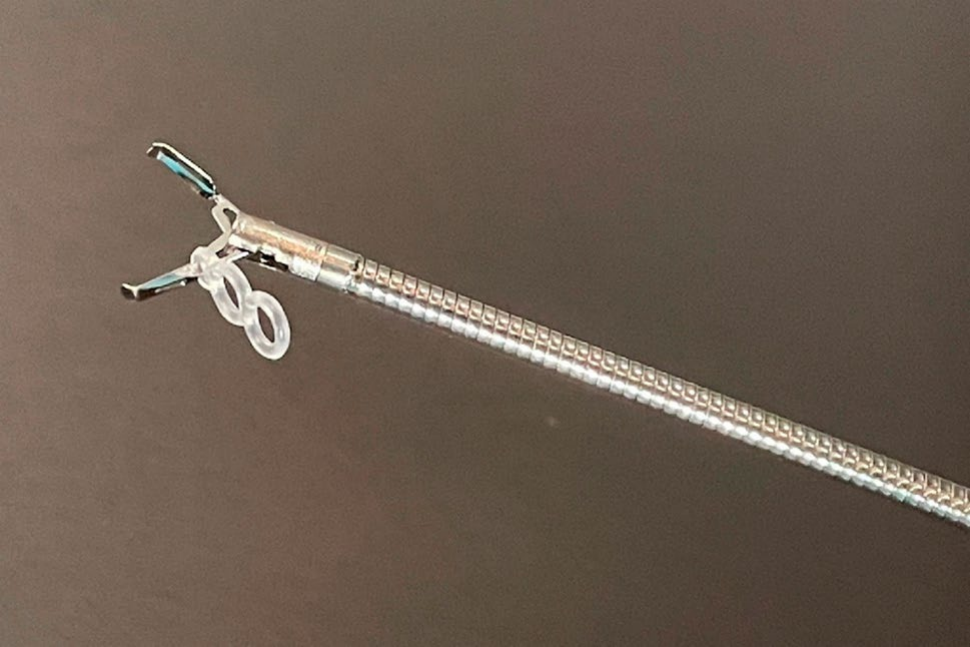
Photo of ETD. ETD is a device equipped with a silicone band attached to a clip. *ETD* Elastic Traction Device

After the ETD was placed on the lesion, the band was grasped with a SureClip (MICRO-TECH, Nanjing, China) and secured to the site opposite the lesion, where traction would be most effective. Traction with an ETD improves the visibility of the submucosal layer and facilitates submucosal dissection. Subsequently, submucosal dissection was performed sequentially. The silicone band of the ETD consisted of two loops, and the direction and tension could be adjusted by applying traction during the dissection process. Dissection continued until the lesion was completely resected en bloc (Fig. 4, Video 1).

### Procedure for the S group

**Fig. 5:**
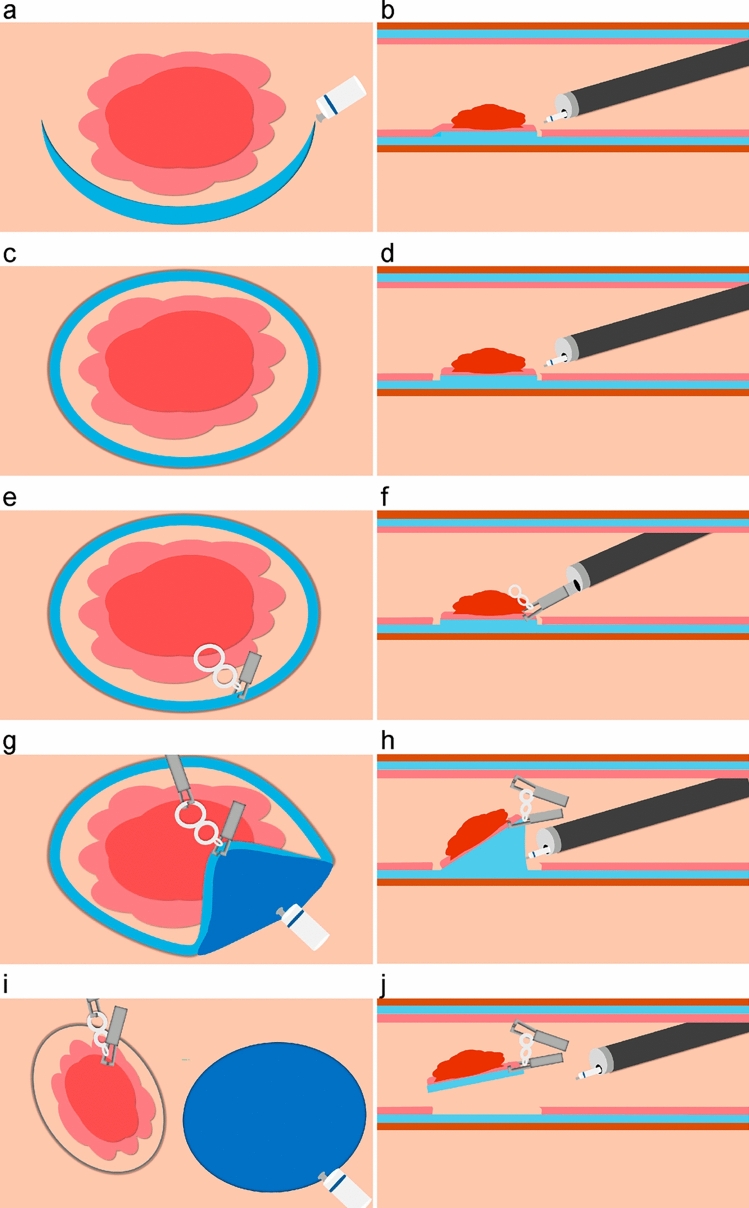
Schematic diagram showing the treatment strategy of the S group. **a, c, e, g, i** From a top view. **b, d, f, h, j** From a side view. **a, b** The initial mucosal incision was made on the anal or oral side of the lesion. **c, d** A circumferential mucosal incision around the lesion and a slight submucosal dissection were performed. **e, f** The ETD was attached. **g, h** The band portion was gripped with a SureClip (MICRO-TECH, Nanjing, China) and fixed at the opposite site. Traction improved the visibility of the submucosal layer and allowed for easier submucosal dissection. **i, j** The dissection was then continued sequentially, leading to the en bloc resection of the lesion. *ETD* Elastic Traction Device

A submucosal injection was administered around the target lesion, followed by a circumferential mucosal incision from the anal or oral side of the lesion. After slight submucosal dissection, ETD was applied. In the schedule (S) group, it was important to attach an ETD as soon as possible. Therefore, submucosal dissection after circumferential mucosal incision was minimized to the extent necessary for ETD placement. Dissection was then continued sequentially, and the lesion was resected en bloc (Fig. 5 and Video 2).

### Procedure for R group

**Fig. 6:**
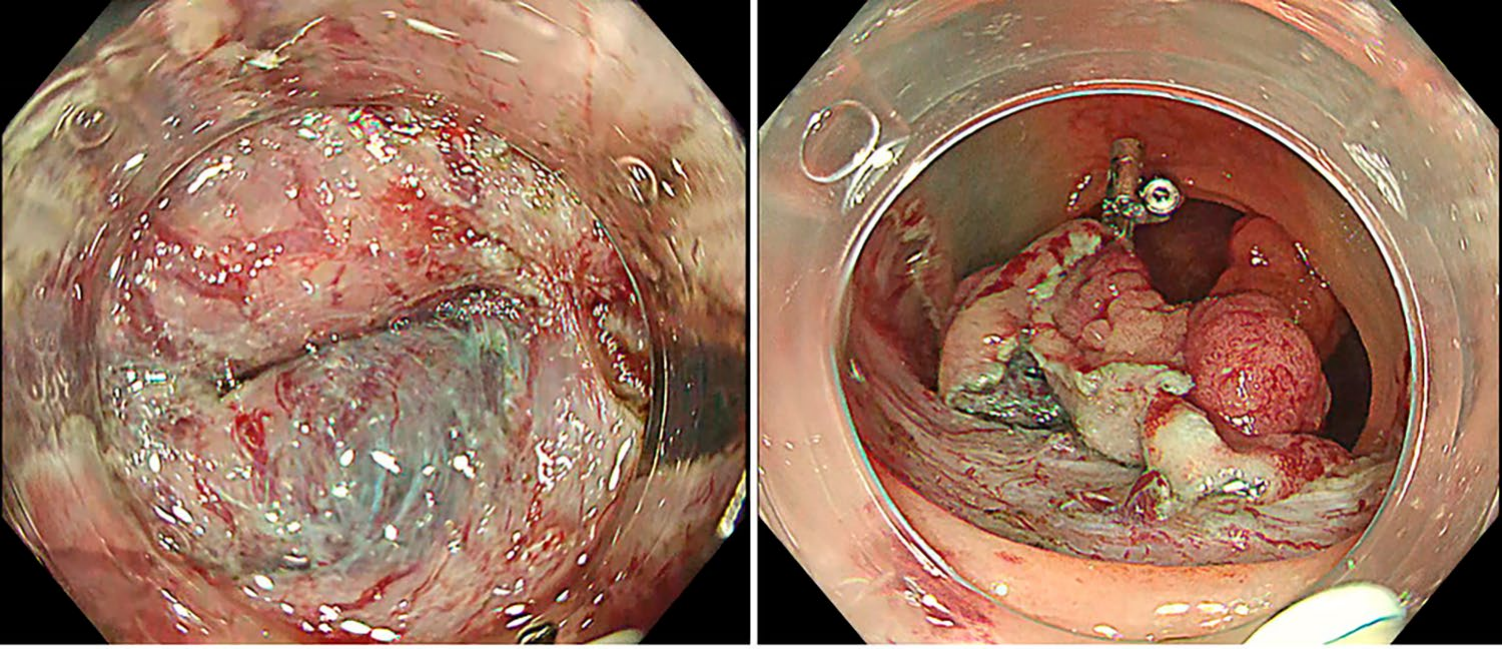
A case from the R group. Owing to significant fibrosis in the submucosal layer, traction using ETD was required. *ETD* Elastic Traction Device

The ETD was applied when the operator determined that it was difficult to continue the procedure without traction owing to factors such as fibrosis and poor maneuverability (Fig. 6). The application and dissection procedures were performed in the same manner as in the S group.

### Definitions

The definitions and calculation methods used in this study are summarized in Table 1.

**Table 1:** Definitions and calculations for colorectal ESD

Definition item	Definition/description
Macroscopic classification	Based on the Paris classification:
	0-I type: protruded
	0-IIa/IIb types: elevated
	0-IIc/0-IIa + IIc types: depressed [14]
Endoscopist classification	Expert: > 100 colorectal ESD case s
	Trainee: < 100 colorectal ESD cases
Procedure time	Time from the start of the mucosal incision to the completion of specimen resection
Specimen area	Calculated assuming an elliptical shape:
	Area = (major axis/2) × (minor axis/2) × π
Dissection speed	Calculated by dividing the specimen area by the dissection time
Calculation of dissection speed in the S group	Before ETD application
	Cut area before ETD placement = 1.5 mm × 2 × (circumference of the specimen) (1.5 mm represents the length of the metal part of the primary knife; assumes a circumferential mucosal incision and one round of submucosal dissection before ETD placement)
	Dissection speed before ETD placement = (cut area before ETD placement)/(time until ETD placement)
	After ETD application
	Dissection speed after ETD placement = (total cut area − cut area before ETD placement) / (time from ETD placement to resection completion)
En bloc resection	Resection of the lesion in one piece without any residual tissue
R0 resection	Histopathological confirmation that both horizontal and vertical margins are negative (HM0, VM0)

*ESD* endoscopic submucosal dissection, *ETD* Elastic Traction Device

### Statistical analysis

Statistical analyses were performed using SAS version 9.4 (SAS Institute, Cary, NC, USA). Continuous variables are presented as means ± standard deviations, while categorical data are expressed as absolute numbers and percentages. Continuous variables were compared using either the independent t test or paired *t* test, as appropriate. Pearson’s chi-squared test or Fisher’s exact test was used to compare categorical variables. Propensity scores for each lesion were calculated using multivariate logistic regression, based on clinically relevant variables associated with the duration of the ESD procedure, including the operator, lesion location, macroscopic type, lesion size, and specimen size. For the comparison between the C-ESD and T-ESD groups, a 1:1 PSM with a greedy matching algorithm was performed using a caliper width equal to 0.2 times the standard deviation of the logit of the propensity score. In the comparison between the S and R groups, a 2:1 PSM was conducted. The standardized difference was used to assess balance after PSM. Statistical significance was set at *p* < 0.05.

## Results

### Comparison between C-ESD and T-ESD groups

Comparison between the C-ESD and T-ESD groups included 136 lesions in the C-ESD group and 124 lesions in the T-ESD group. After PSM, 101 lesions were matched in each group. The lesion backgrounds of the T-ESD and C-ESD groups are shown in Table 2. No significant differences were observed in the clinicopathological features of the resected lesions between the two groups before or after matching (Table 3). A comparison of the treatment outcomes is presented in Table 4.

**Table 2:** Comparison of patient backgrounds between the C-ESD and T-ESD groups

	Before matching		*p* value	SD	After matching		*p* value	SD
	C-ESD (*n* = 136)	T-ESD (*n* =124)			C-ESD (*n* =101)	T-ESD (*n* =101)		
Age (years), average	71.37 ± 10.54	69.67 ± 9.33	0.17	0.171	72.03 ± 10.22	69.14 ± 9.11	0.03	0.29
Operator, *n* (%)			0.19				0.61	
Expert	108 (79.4)	90 (72.6)		0.158	79 (78.2)	76 (75.2)		0.071
Trainee	28 (20.6)	34 (27.4)		− 0.158	22 (21.8)	25 (24.8)		− 0.071
Sex,* n* (%)			0.76				0.55	
Male	88 (64.7)	78 (62.9)		0.038	66 (65.3)	62 (61.4)		0.081
Female	48 (35.3)	46 (37.1)		− 0.038	35 (34.7)	39 (38.6)		− 0.081
Tumor location,* n* (%)			0.0069				0.63	
Right colon	68 (50.0)	83 (66.9)	< 0.01	− 0.35	60 (59.4)	66 (65.3)	0.47	− 0.122
Left colon	26 (19.1)	22 (17.7)	0.87	0.036	18 (17.8)	17 (16.8)	1	0.026
Rectum	42 (30.9)	19 (15.3)	< 0.01	0.38	23 (22.8)	18 (17.8)	0.48	0.125
Morphology,* n* (%)			0.0011				0.98	
Protruded	51 (37.5)	21 (16.9)	< 0.01	0.48	22 (21.8)	21 (20.8)	0.86	− 0.024
Elevated	73 (53.7)	88 (71.0)	< 0.01	− 0.36	67 (66.3)	68 (67.3)	0.88	0.021
Depressed	12 (8.8)	15 (12.1)	0.39	− 0.14	12 (11.9)	12 (11.9)	1	0
Resected tumor size (average, mm)	30.10 ± 8.39	29.84 ± 7.92	0.8	0.032	30.26 ± 8.45	29.88 ± 7.96	0.74	0.046
Resected specimen size (average, mm)	39.85 ± 9.97	40.25 ± 8.91	0.73	− 0.042	40.75 ± 9.99	40.04 ± 8.73	0.58	0.076

*C-ESD* conventional endoscopic submucosal dissection, *T-ESD* traction endoscopic submucosal dissection, *SD* standard deviation

**Table 3:** Clinicopathological features of resected specimens in the C-ESD and T-ESD groups

	Before matching			After matching		
	C-ESD (*n* = 136)	T-ESD (*n* = 124)	*p* value	C-ESD (*n* = 101)	T-ESD (*n* = 101)	*p* value
Histology,* n* (%)			0.42			0.58
Adenoma	60 (44.1)	46 (37.1)		43 (42.6)	36 (35.6)	
Intramucosal cancer	51 (37.5)	53 (42.7)		38 (37.6)	42 (41.6)	
Submucosal invasive cancer	15 (11.0)	19 (15.3)		12 (11.9)	17 (16.8)	
Sessile serrated lesions	10 (7.4)	6 (4.8)		8 (7.9)	6 (5.9)	

*C-ESD* conventional endoscopic submucosal dissection, *T-ESD* traction endoscopic submucosal dissection, *SD* standard deviation

**Table 4:** Comparison of treatment outcomes between the C-ESD and T-ESD groups

	Before matching			After matching		
	C-ESD (*n* = 136)	T-ESD (*n* = 124)	*p* value	C-ESD (*n* = 101)	T-ESD (*n* = 101)	*p* value
Procedure time (average, min)	43.47 ± 34.02	53.11 ± 37.84	0.03	47.13 ± 36.87	52.96 ± 39.17	0.27
Dissection speed (average, mm^2^/min)	33.77 ± 21.43	28.51 ± 18.87	0.03	33.54 ± 21.38	28.38 ± 18.81	0.07
Adverse events,* n* (%)						
Muscle layer damage	7 (5.1)	11 (8.9)	0.23	6 (5.9)	9 (8.9)	0.42
Perforation	4 (2.9)	8 (6.5)	0.17	4 (4.0)	6 (5.9)	0.52
Delayed bleeding	0	1(0.8)	0.8	0	1(1.0)	0.32
En bloc resection rate,* n* (%)	136 (100.0)	120 (96.8)	0.03	101 (100.0)	99 (98.0)	0.16
R0 resection rate,* n* (%)	132 (97.1)	114 (91.9)	0.06	97 (96.0)	94 (93.1)	0.35

*C-ESD* conventional endoscopic submucosal dissection, *T-ESD* traction endoscopic submucosal dissection

After applying the PSM method, no significant differences were observed in procedure time, dissection speed, en bloc resection rate, R0 resection rate, muscle layer injury rate, perforation rate, or postprocedural bleeding rate.

### Comparison between S group and R group

For comparison between the S and the R groups, the analysis included 88 and 36 patients in the S and R groups, respectively. After PSM, 58 and 33 patients from groups S and R, respectively, were matched.

A comparison of the lesion backgrounds is shown in Table 5. No significant differences were found in the clinicopathological features of the resected lesions between the two groups before or after matching (Table 6). A comparison of the treatment outcomes is presented in Table 7. The procedure time was significantly shorter in the S group (S group: 43.93 ± 36.30 min vs. R group: 70.30 ± 41.38 min, *p* < 0.01), and the dissection speed was significantly faster in the S group (S group: 35.80 ± 21.82 mm^2^/min vs. R group: 20.23 ± 10.81 mm^2^/min, *p* < 0.01) after PSM method. No significant differences were observed in the en bloc resection, R0 resection, muscle layer injury, perforation, or postprocedural bleeding rates after PSM (en bloc resection rate: S group, 96.6% vs. R group, 93.9%, *p* = 0.56; R0 resection rate: S group, 91.4% vs. R group, 87.9%, *p* = 0.59; muscle layer injury rate: S group, 6.9% vs. R group, 18.2%, *p* = 0.1; perforation rate: S group, 3.4% vs. R group, 12.1%, *p* = 0.11; postprocedural bleeding rate: S group, 1.7% vs. R group, 0%, *p* = 0.52).

**Table 5:** Comparison of patient backgrounds between the schedule and the rescue groups

	Before matching		*p* value	SD	After matching		*p* value	SD
	Schedule (*n* = 88)	Rescue (*n* = 36)			Schedule (*n* = 58)	Rescue (*n* = 33)		
Age (years), average	70.40 ± 8.77	67.89 ± 10.50	0.17	0.26	72.03 ± 8.36	67.64 ± 9.75	0.02	0.48
Operator,* n* (%)			0.2				0.86	
Expert	61 (69.3)	29 (80.6)		− 0.26	40 (76.9)	22 (78.6)		− 0.04
Trainee	27 (30.7)	7 (19.4)		0.26	12 (23.1)	6 (21.4)		0.04
Sex,* n* (%)			0.07				0.01	
Male	51 (58.0)	27 (75.0)		− 0.37	29 (50.0)	25 (75.8)		− 0.55
Female	37 (42.0)	9 (25.0)		0.37	29 (50.0)	8 (24.2)		0.55
Tumor location,* n* (%)			0.07				0.86	
Right colon	56 (63.6)	27 (75.0)		− 0.25	45 (86.5)	23 (82.1)		0.12
Left colon	20 (22.7)	2 (5.6)		0.51	3 (5.8)	2 (7.1)		− 0.05
Rectum	12 (13.6)	7 (19.4)		− 0.16	4 (7.7)	3 (10.7)		− 0.1
Morphology,* n* (%)			0.6				0.87	
Protruded	13 (14.8)	8 (22.2)		− 0.19	7 (13.5)	5 (17.9)		− 0.12
Elevated	64 (72.7)	24 (66.7)		0.13	39 (75.0)	20 (71.4)		0.08
Depressed	11 (12.5)	4 (11.1)		0.04	6 (11.5)	3 (10.7)		0.03
Resected tumor size (average, mm)	29.33 ± 7.51	31.08 ± 8.83	0.26	− 0.21	30.62 ± 7.69	30.42 ± 8.46	0.91	0.02
Resected specimen size (average, mm)	39.34 ± 8.20	42.47 ± 10.22	0.07	− 0.34	41.36 ± 8.15	41.27 ± 9.04	0.96	0.01

*SD* standard deviation

**Table 6:** Clinicopathological features of resected specimens in the schedule and rescue groups

	Before matching			After matching		
	Schedule (*n* = 88)	Rescue (*n* = 36)	*p* value	Schedule (*n* = 58)	Rescue (*n* = 33)	*p* value
Histology,* n* (%)			0.09			0.97
Adenoma	35 (39.8)	11 (30.6)		24 (41.4)	11 (33.3)	
Intramucosal cancer	39 (44.3)	14 (38.9)		25 (43.1)	13 (39.4)	
Submucosal invasive cancer	9 (10.2)	10 (27.8)		6 (10.3)	9 (27.3)	
Sessile serrated lesions	5 (5.7)	1 (2.8)		3 (5.2)	0 (0.0)

**Table 7:** Comparison of treatment outcomes between the schedule group and the rescue groups

	Before matching		*p* value	After matching		*p* value
	Schedule (*n* = 88)	Rescue (*n* = 36)		Schedule (*n* = 58)	Rescue (*n* = 33)	
Procedure time (average, min)	44.99 ± 33.56	72.97 ± 40.77	< 0.01	43.93 ± 36.30	70.30 ± 41.38	< 0.01
Dissection speed (average, mm^2^/min)	31.85 ± 20.45	20.32 ± 10.69	< 0.01	35.80 ± 21.82	20.23 ± 10.81	< 0.01
Adverse events,* n* (%)						
Muscle layer damage	5 (5.7)	6 (16.7)	0.05	4 (6.9)	6 (18.2)	0.1
Perforation	4 (4.5)	4 (11.1)	0.17	2 (3.4)	4 (12.1)	0.11
Delayed bleeding	1 (1.1)	0 (0.0)	0.52	1 (1.7)	0 (0.0)	0.45
En bloc resection rate,* n* (%)	86 (97.7)	34 (94.4)	0.35	56 (96.6)	31 (93.9)	0.56
R0 resection rate,* n* (%)	83 (94.3)	31 (86.1)	0.12	5 (8.6)	4 (12.1)	0.59

Additionally, in the S group, a comparison of the dissection speed before and after ETD attachment showed a significant improvement in dissection speed after ETD attachment (before attachment: 22.22 mm^2^/min vs. after attachment: 49.59 mm^2^/min, *p* < 0.001).

## Discussion

This study did not find significant improvements in procedural time or dissection speed with the use of the ETD in colorectal ESD. However, it suggested that planned and early ETD application enhances procedure time and dissection speed.

Traction devices in colorectal ESD, such as the S–O clip traction and clip-and-thread methods, have been shown to reduce procedure time and improve en bloc resection rates [12, 15–17]. The S–O clip is a traction device consisting of a metal clip, a 5 mm spring, and a nylon loop. The device can be applied through the forceps opening and does not require the removal of the endoscope. Traction with an S–O clip is expected to improve the visibility of the submucosal layer and enable rapid dissection.

The ETD is a new traction device with a double elastic ring attached to a clip. Similar to the S–O clip, the ETD is a traction device that can be applied through a forceps opening without removing the endoscope. The use of elastic rings for traction in ESD has been reported to offer advantages such as ease of operation and reduced risk of tissue damage due to excessive traction. Traction with an elastic ring may fully expose the submucosa and reduce the risk of bleeding and perforation [7].

In this study, there were no significant differences in the procedure time or dissection speed was not significantly different between the traction and non-traction groups. Previous studies have reported that the use of traction devices improves the procedure time and dissection speed in colorectal ESD [12, 15, 16]. However, these studies involved fewer surgeons performing ESD than the current study, potentially reflecting the effect of the learning curve, or are prospective studies with limited case numbers. Notably, our institution is a high-volume center with mature expert workmanship, suggesting a stabilized learning curve. In addition, our data indicate that, compared with the C-ESD group, the T-ESD group had a significantly higher proportion of lesions located in the right colon and with a protruded morphology, both known to increase technical difficulty [18, 19]. These findings suggest that the ETD may have been primarily used to shorten procedure time as well as ensure a safe dissection in these challenging cases. Therefore, direct comparison with our study is challenging. Furthermore, few studies have examined the efficient use of traction devices and the timing of their application. This is the first study to examine the efficient use of a novel traction device, the ETD, for colorectal ESD.

The first advantage of the ETD is that the clip can be easily rotated, opened, and closed to ensure that the device is securely implanted without grasping the muscle layer. Furthermore, it allows easy regrasping of lesions. These features facilitate the optimal placement of a lesion to maximize the effectiveness of the traction device. Second, the ETD enables retraction and adjustment of the traction direction as needed. Even if initial traction is sufficient, it may become inadequate during dissection, or manipulation might become challenging owing to peristalsis. The dual silicone bands of the ETD allow for easy adjustment of the direction and strength of traction in such situations. Third, they are straightforward to set up. For instance, the clip-and-thread method can be easily applied to rectal lesions, although the procedure for proximal colorectal lesions is more complex [17]. ETD features a preinstalled silicone ring and requires no special procedures before application.

In this study, before adjusting for PSM, the T-ESD group performed significantly worse than the C-ESD group in terms of procedure time, dissection speed, and en bloc resection rate. In the T-ESD group, more cases were performed by experts and thus more difficult cases may have been included, possibly leading to selection bias in this retrospective study.

Comparing the S and R groups, the S group exhibited significantly shorter procedure times and faster dissection speeds, which remained consistent after PSM. In actual ESD procedures where the submucosal flap is small, difficulties often arise because of limited scope maneuverability and submucosal visibility [20, 21]. Early ETD placement facilitates access beneath the lesion into the submucosa, enhancing visibility and maneuverability, thus increasing treatment efficiency. Furthermore, comparing dissection speeds before and after ETD application within the S group revealed a significant acceleration post-application, corroborating the result that early ETD use enhances dissection speed.

Complication rates were somewhat higher in the T-ESD group than in the C-ESD group, although these differences were not statistically significant. Additionally, there were no significant differences in outcomes when comparing procedures performed by trainees and experts (muscle layer injury: 5/34 [14.71%] in trainees versus 6/90 [6.67%] in experts, *p* = 0.16; perforation: 2/34 [5.88%] in trainees versus 6/90 [6.67%] in experts, *p* = 0.87; delayed bleeding: 0/34 [0%] in trainees versus 1/90 [1.11%] in experts, *p* = 0.53). We suspect that this might partly be due to ETD being used more frequently in potentially challenging cases to facilitate safe dissection; however, this possibility was not definitively demonstrated in our study. Further prospective research is warranted to determine whether ETD use ultimately leads to better patient outcomes.

This study had several limitations. First, it was a single-center retrospective study with a limited number of cases. In particular, the number of cases in group R was small, indicating that a prospective study with a larger sample size is needed to obtain more accurate results. In addition, this study did not include a power analysis and may not have had a sufficient number of cases to adequately evaluate the usefulness of ETD. Second, treatment strategies were left to the discretion of the endoscopists, which may have introduced a selection bias. Third, there may have been inaccuracies in the measurement of the procedure time. During the procedures, operator changes were made for safety reasons (i.e., one endoscopist was replaced by another when necessary), which may have affected the accuracy of the recorded procedure times. Finally, factors that increase technical difficulty, such as fibrosis, were not adequately considered, and the overall assessment of case difficulty was incomplete. Future studies should consider these factors to further clarify the clinical utility of the ETD.

## Conclusion

The ETD did not consistently shorten procedure time or improve dissection speed; however, its planned and early use may offer potential benefits. Further prospective, multicenter studies are needed to determine its optimal role and clinical value.

## Supplementary Information

Below is the link to the electronic supplementary material.

Video 1 ETD-assisted colorectal endoscopic submucosal dissection performed for a 36 mm lesion in the ascending colon. Traction using the ETD improved the visibility of the submucosal layer and facilitated easier submucosal dissection. In this case, additional traction was applied using dual ETD rings (MP4 48010 KB)

Video 2 A 40 mm lesion in the cecum of the scheduled group. After performing a circumferential mucosal incision, the ETD was promptly applied (MP4 29415 KB)

Supplementary file3 (DOCX 37 KB)
